# Diversity analysis of leaf endophytic fungi and rhizosphere soil fungi of Korean Epimedium at different growth stages

**DOI:** 10.1186/s40793-022-00446-w

**Published:** 2022-10-21

**Authors:** Chen Jiawen, Wu Yuan, Zhuang Xin, Guo Junjie, Hu Xing, Xiao Jinglei

**Affiliations:** Institute of Identification Department of Traditional Chinese Medicine, Changchun University of Traditional Chinese Medicine, Changchun, Jin Lin Province China

**Keywords:** *Epimedium koreanum *Nakai, Endophytic fungi, Rhizosphere soil, Community diversity, *Sebacina sp.*

## Abstract

**Background:**

Rhizosphere fungi and endophytic fungi play key roles in plant growth and development; however, their role in the growth of *Epimedium koreanum* Nakai at different stages remains unclear. Here, we used the Illumina MiSeq system, a high-throughput sequencing technology, to study the endophytic fungi and rhizosphere microbiome of Korean Epimedium.

**Results:**

*Epimedium koreanum *Nakai rhizosphere soil and leaves had highly diverse fungal communities during the growth process. The relative abundance of soil fungi in the rhizosphere stage was higher than that of leaf endophytic fungi in the early growth stage, but the overall abundance was basically equal. *Sebacina* is a significantly divergent fungal genera, and *Sebacina sp.* are present among leaf fungi species in the rhizosphere soil of *Epimedium koreanum *Nakai*. Sebacina sp*. can move to each other in rhizosphere soil fungi and leaf endophytes. VIF (variance inflation factor) analysis showed that soluble salt, whole nitrogen, alkaline lysis nitrogen, whole phosphorus, total potassium, and fast-acting potassium are useful environmental factors for rhizosphere soil and leaf endophytic fungi: potassium, total nitrogen, whole phosphorus, and three environmental factors were significantly and positively associated with the relative abundance of *Sebacina sp.*

**Conclusions:**

(1) This study is the first to clarify the species diversity of fungi in *Epimedium koreanum* Nakai leaf and rhizosphere soil. (2) Different fungal communities of rhizosphere soil fungi and leaf endophytic fungi at different growth stages of *Epimedium koreanum* Nakai were examined. (3) *Sebacina sp*. can move to each other between rhizosphere soil fungi and leaf endophytic fungi. (4) Nitrogen, phosphorus and potassium elements in the environment have a significant positive effect on the relative abundance of *Sebacina sp.*

**Supplementary Information:**

The online version contains supplementary material available at 10.1186/s40793-022-00446-w.

## Background

Rhizosphere is the soil attached to or located in close proximity to plant roots, and is known as a hotspot of microbial activity and diversity. Understanding the taxonomic and functional components of the rhizosphere microbiome and how they differ from those of the bulk soil microbiome is crucial, so that they can be manipulated for establishing a well-functioning sustainable ecosystem [1]. Fungi are one of the highly diverse and active groups of microbes in the rhizosphere, which include species that have positive as well as adverse effects on plant growth, nutrition and health [2]. Plants can actively shape and select their rhizosphere mycobiome by secreting photosynthates and root exudates [3]. The amount and type of plant root exudates as well as the morphology of plant roots are species-specific [4, 5], and contribute to the effect of plant species identity on fungal communities [6, 7]. The surrounding bulk soil (i.e., soil not in close contact with plant roots) serves as a propagule bank of fungi that can potentially be selected by the growing plant roots [8]. The diversity, composition and functionality of fungal communities also are affected by soil–plant compartments [9]. The plant proactively recruits microbes by releasing exudates from its roots [10, 11]. These microbes can be further transferred to berries, thus profoundly impacting berry quality [12–14]. Therefore, changes in the rhizosphere soil fungal community affects crop yield and quality.

Fungal endophytes commonly infect host plants asymptomatically, and reside within the tissues of living plants [15]. Some endophytic fungi increase the fitness of host plants by enhancing their resistance to abiotic [16] and biotic [17] stresses. Currently, considerable research is being conducted on the biodiversity, chemistry and metabolite bioactivity of endophytic fungi to understand the relationship between endophytes and their host plants [18]. While there is little information on whether culturable endophytic communities change seasonally, the available evidence suggests that this might occur in both the roots and leaves of plants [19]. Jin et al. [20] showed that the total colonization frequency and species richness of endophytic fungi are higher in roots than in leaves and stems of *Stellera chamaejasme* L. in northwestern China. In addition, they found that the structure of fungal communities in plant tissues differed significantly between the stages of leaf emergence and dormancy. Zeng et al. [21] showed that temporal variation in the diversity of root endophytic fungi of *Bletilla striata* was consistent with the degree of enrichment at 13 °C, which also met the nutritional requirements of the plant. Thus, it can be concluded that there is some regularity in the changing trend of endophytic and rhizosphere soil fungal communities at different stages of plant growth.

Based on the above research results, we hypothesize that: (1) the community structure of endophytic fungi in the rhizosphere soil and leaves of *Epimedium koreanum *Nakai exhibit a wane-and-wax trend; (2) different types of environmental factors determine the rhizosphere microbial community to different degrees; and (3) a certain fungal community may emerge or disappear in the plant rhizosphere or leaves.

## Methods

### Study site and sample collection

Five *Epimedium koreanum *Nakai leaf samples (MY1–5) at different growth stages (20 days apart) were collected from May 10 to August 1, 2020 in Ant He Town, Linjiang City, Jilin Province, China. During the same time period, the rhizosphere soil samples of *Epimedium koreanum *Nakai were collected using the "shaking sampling method". Briefly, after sampling, large pieces of soil at the root were removed, and the samples were transported to the laboratory on ice. Then, loose soil was removed from the roots by shaking, and residual soil on the roots was removed with a sterile brush. Subsequently, soil samples collected from the same square were mixed in equal quantities, flash-frozen in liquid nitrogen and stored in a freezer at − 80 °C. The rhizosphere soil fungi (MT1–5) groups were processed similarly.

### DNA extraction

Total community genomic DNA extraction was performed using a E.Z.N.A.Soil DNA Kit (Omega, M5635-02, USA), following the manufacturer’s instructions. We measured the concentration of the DNA using a Qubit 4.0(Thermo, USA) to ensure that adequate amounts of high-quality genomic DNA had been extracted.

### 16S rRNA gene amplification by PCR

Our target was the V3–V4 hypervariable region of the fungi 16S rRNA gene. PCR was started immediately after the DNA was extracted.The 16S rRNA V3–V4 amplicon was amplified using 2 × Hieff^®^ Robust PCR Master Mix (Yeasen, 10105ES03, China). Two universal fungi 16S rRNA gene amplicon PCR primers (PAGE purified) were used: the amplicon PCR forward primer (CCTACGGGNGGCWGCAG) and amplicon PCR reverse primer (GACTACHVGGGTATCTAATCC). The reaction was set up as follows: microbial DNA (10 ng/μl) 2 μl; amplicon PCR forward primer (10 μM) 1 μl; amplicon PCR reverse primer (10 μM) 1 μl; 2 × Hieff^®^ Robust PCR Master Mix (Yeasen, 10105ES03, China) (total 30 μl). The plate was sealed and PCR performed in a thermal instrument (Applied Biosystems 9700, USA) using the following program: 1 cycle of denaturing at 95 °C for 3 min, first 5 cycles of denaturing at 95 °C for 30 s, annealing at 45 °C for 30 s, elongation at 72 °C for 30 s, then 20 cycles of denaturing at 95 °C for 30 s, annealing at 55 °C for 30 s, elongation at 72 °C for 30 s and a final extension at 72 °C for 5 min. The PCR products were checked using electrophoresis in 1% (w/v) agarose gels in TBE buffer (Tris, boric acid, EDTA) stained with ethidium bromide (EB) and visualized under UV light.

### 16S rRNA gene library construction, quantification, and sequencing

We used AMPure XP beads to purify the free primers and primer dimer species in the amplicon product. Samples were delivered to Sangon BioTech (shanghai) for library construction using universal Illumina adaptor and index. Before sequencing, the DNA concentration of each PCR product was determined using a Qubit^®^ 4.0 Green double-stranded DNA assay and it was quality controlled using a bioanalyzer (Agilent 2100, USA). Depending on coverage needs, all libraries can be pooled for one run.The amplicons from each reaction mixture were pooled in equimolar ratios based on their concentration. Sequencing was performed using the Illumina MiSeq system (Illumina MiSeq, USA), according to the manufacturer’s instructions.

After sequencing, the two short Illumina readings were assembled by PEAR software (version 0.9.8) according to the overlap and fastq files were processed to generate individual fasta and qual files, which could then be analyzed by standard methods. The effective tags were clustered into operational taxonomic units (OTUs) of ≥ 97% similarity using Usearch software (version 11.0.667). Chimeric sequences and singleton OTUs (with only one read) were removed, after which the remaining sequences were sorted into each sample based on the OTUs. The tag sequence with the highest abundance was selected as a representative sequence within each cluster. fungal OTU representative sequences was classified taxonomically by blasting against the RDP Database and UNITE fungal ITS Database, respectively.

### Statistical analysis

The α-diversity indices (including Chao1, Simpson, and Shannon indices) were quantified in terms of OTU richness. To assess sample adequacy, rarefaction curves of the observed numbers of OTUs were constructed. All α diversity indices were calculated with Mothur software (version 3.8.31) [22]. The OTU rarefaction curve and rank abundance curves were plotted in R (version 3.6.0). To estimate the diversity of the microbial community of the sample, we calculated the withinsample (alpha) diversity by T test for two groups and multiple group comparisons were made using ANOVA [23] test. Beta diversity evaluates differences in the microbiome among samples and is normally combined with dimensional reduction methods such as principal coordinate analysis (PCoA), non-metric multidimensional scaling (NMDS), or constrained principal component analysis (PCA) to obtain visual representations. These analyses were visualized using R vegan package (version 2.5–6), and finally the inter-sample distances were presented as scatterplots. Difference comparison is used to identify features with significantly different abundances between groups using STAMP (version2.1.3) [23], LEfSe (version1.1.0) [24]] and GraPhlAn software version (1.1.3) [23]. Correlation coefficients and *p*-values between communities/OTUs were calculated using SparCC (version 1.1.0) [25], and correlation matrix heatmaps were drawn using R corrplot package (version 0.84) [26]. R ggraph package (version 2.0.0) is used to build network graphs.

### Environmental factor determination

The experimental method of soil composition detection refers to the Soil Agricultural Chemical Analysis Method [27]. The indexes selected include soil pH value, soluble salt, organic matter, total nitrogen, whole phosphorus, total potassium, alkali solution nitrogen, effective phosphorus and quick-acting potassium.

## Results

### Species annotation and alpha diversity analysis

**Fig. 1:**
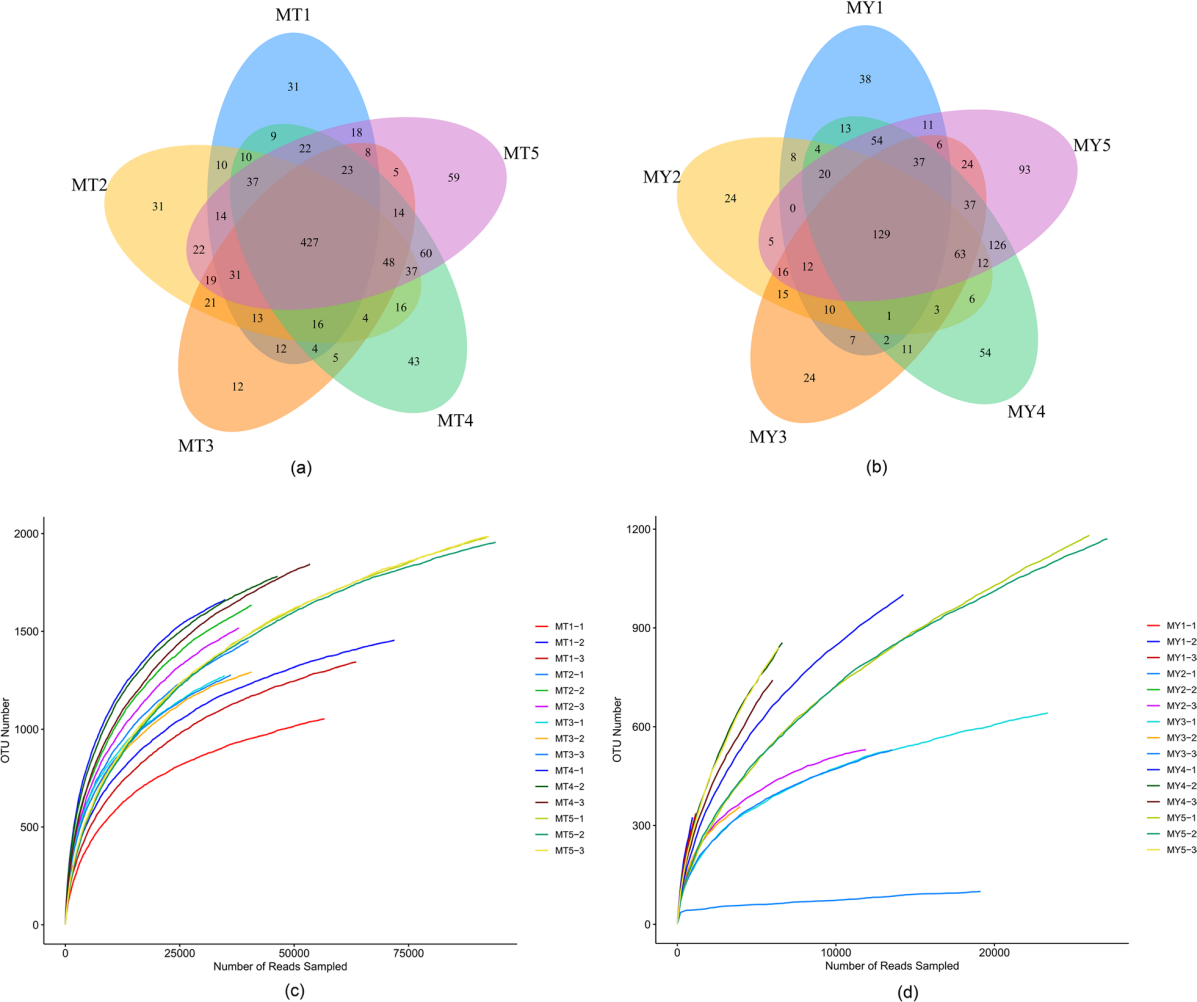
**a** and **b** Venn diagrams at the OTU level in MT (**a**) and MY (**b**) groups. **c** and **d** Dilutional plots of MT (**c**) and MY (**d**) groups

A total of ten composite samples, including five rhizosphere soil fungi (MT1–5) and five leaf endophytic fungi (MY1–5) samples (with three biological replicates per sample), were subjected to high-throughput sequencing using the Illumina MiSeq system. A total of 1,700,991 raw reads, including 987,902 reads from rhizosphere soil fungi (average: 197,580 reads per sample) and 711,398 reads from leaf endophytic fungi (average: 142,279 reads per sample). Based on the least number of sequences detected among the assayed samples, we randomly selected and compared 288,425 and 229,301 reads of rhizosphere soil fungi and leaf endophytic fungi per sample. A total of 674 operational taxonomy units (OTUs) were identified in five groups of rhizosphere soil fungi (MT1–5), accounting for 13.71% of the total OTUs. MT1, MT2, MT3, MT4 and MT5 contained 310, 297, 145, 447 and 475 OTUs, respectively (Fig. 1a). Additionally, 96 OTUs were identified in five groups of leaf endophytic fungi (MY1–5), accounting for 3.27% of the total OTUs. MY1, MY2, MY3, MY4 and MY5 contained 217, 104, 165, 384 and 548 OTUs, respectively (Fig. 1b). The OTUs obtained from all 10 groups of samples were annotated from the phylum to the genus level. Excluding the unclassified OTUs, a total of 935 genera belonging to 341 families, 57 classes and 16 phyla were identified among rhizosphere soil fungi, and a total of 935 genera belonging to 341 families, 154 orders, 57 classes and 16 phyla were identified among the leaf endophytic fungi.

To determine whether the amount of sequencing data generated was reasonable we constructed dilution curves by plotting the amount of extracted data (y-axis) against the alpha diversity index (x-axis), which reflects the abundance and diversity of a given species in the sample (Fig. 1c and d). The alpha diversity index was determined using the Mothur software; the number of OTUs in the community was estimated using ACE and Chao1 indices; microbial diversity in samples was determined using the Shannon index, which is often used along with the Simpson diversity index to reflect the alpha diversity index. The larger the value of the Shannon index, the higher the community diversity. Conversely, the greater the Simpson index, the lower the community diversity. Values of ACE, Shannon and Chao1 indices indicated that the diversity, richness and OTU number of microorganisms in the rhizosphere soil first increased and then decreased at the early stage of *Epimedium koreanum *Nakai growth, whereas those of leaf endophytic fungi first decreased and then increased (Additional file 1: Tables S1 and S2).

### Beta diversity and phylogenetic information visualization

**Fig. 2:**
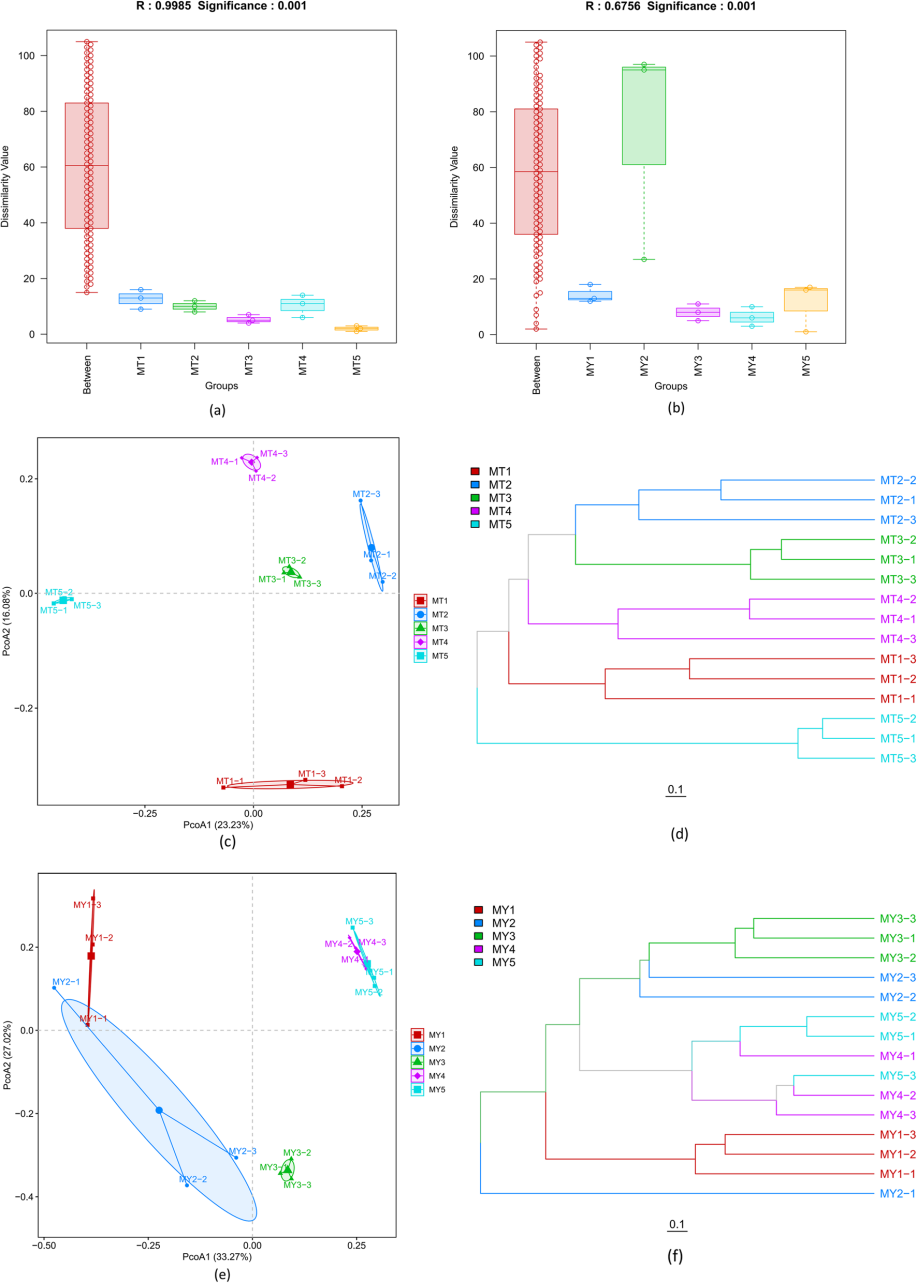

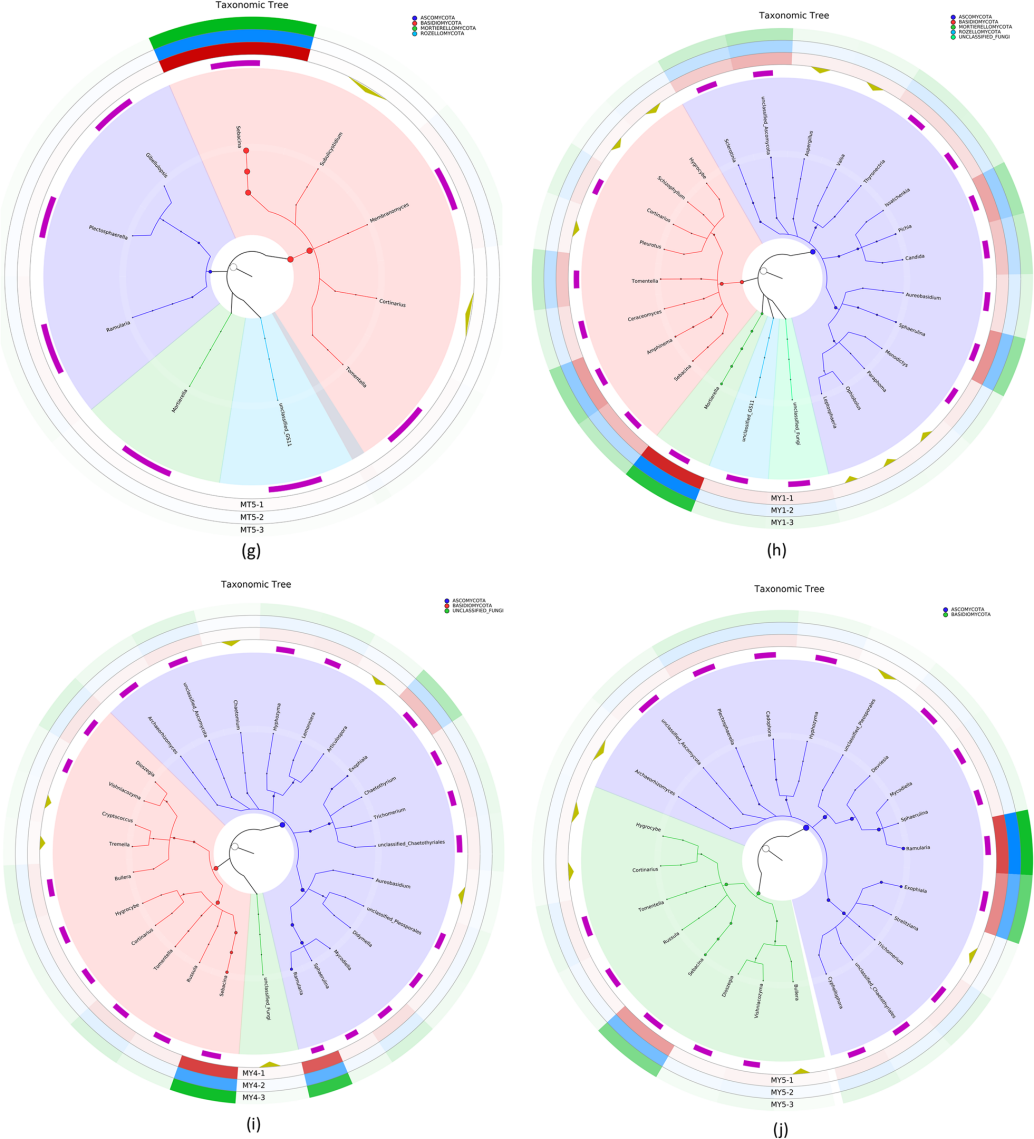
**a** and **b** ANOSIMs of MT (**a**) and MY (**b**) groups. **c** and **d** PCoA (**c**) and hierarchical clustering (**d**) plots of MT groups. **e** and **f** PCoA (**e**) and hierarchical clustering (**f**) plots of MY groups. **g–j** Phylogenetic visualization analysis of MT5 (**g**), MY3 (**h**), MY4 (**i**) and MY5 (**j**) samples using GraPhlAn

Analysis of similarities (ANOSIM) is a non-parametric test used to test whether the differences between groups (two or more) are significantly greater than those within a group. The smaller the *P*-value, the greater the significance of the differences between groups. A statistically significant *P*-value (*P* < 0.05) indicates that the grouping is meaningful. The results of ANOSIM showed that the *P*-values of the two groups of samples were less than 0.5, indicating inter-group differences (Fig. 2a and b).

We evaluated beta diversity at the level of OTUs, which were defined based on a similarity cut-off of 97%. To compare the composition of the identified microbial communities within different plant compartments, hierarchical clustering was performed at the OTU level based on Bray–Curtis dissimilarity. Principal coordinate analysis (PCoA) of MT samples showed that all five groups were dispersed (Fig. 2c). The hierarchical clustering tree (Fig. 2d) showed that when the distance was 0.1, the MT2 and MT3 groups clustered together in one branch, while the remaining three groups (MT1, MT4 and MT5) clustered into separate branches. The PCoA of MY samples (Fig. 2e) revealed an overlap between the components of MY1 and MY2 and between those of MY4 and MY5, and this result was confirmed by hierarchical clustering (Fig. 2f). The MY3 group formed a separate branch, and its components showed no similarity with those of the other groups.

Phylogenetic visualization analysis of the similarity among the MT samples showed that all five groups of samples clustered into a single group at the genus level at the MT5 stage, and into a group at the other stages, indicating that the fungal microbial community in the rhizosphere soil of *Epimedium koreanum *Nakai changed significantly at the late growth stage compared with the early growth stage. *Sebacina sp.* was found at all stages, but its abundance increased significantly at the MT5 stage (Fig. 2g). Phylogenetic visualization analysis of the similarity among the MY samples showed that the five groups of samples could be divided, based on the community structure of endophytic fungi, into two clusters: one containing MY1 and MY2, and the other containing MY3, MY4 and MY5 (Fig. 2h–j). This indicates that the endophytic fungal communities in the first two periods of MY1 and MY2 were similar to each other but distinct from the endophytic fungal communities in the last three periods (MY3–5), which were relatively similar to each other. This shows that the community structure of endophytic fungi of *Epimedium koreanum *Nakai. varied greatly among the different growth stages. The genus *Ramularia* showed the lowest relative abundance in MY1 and the highest relative abundance in MY4 and MY5, while the genus *Sphaerulina* was found in all periods.

### Correlation between the relative abundance of dominant species and collinearity

**Fig. 3:**
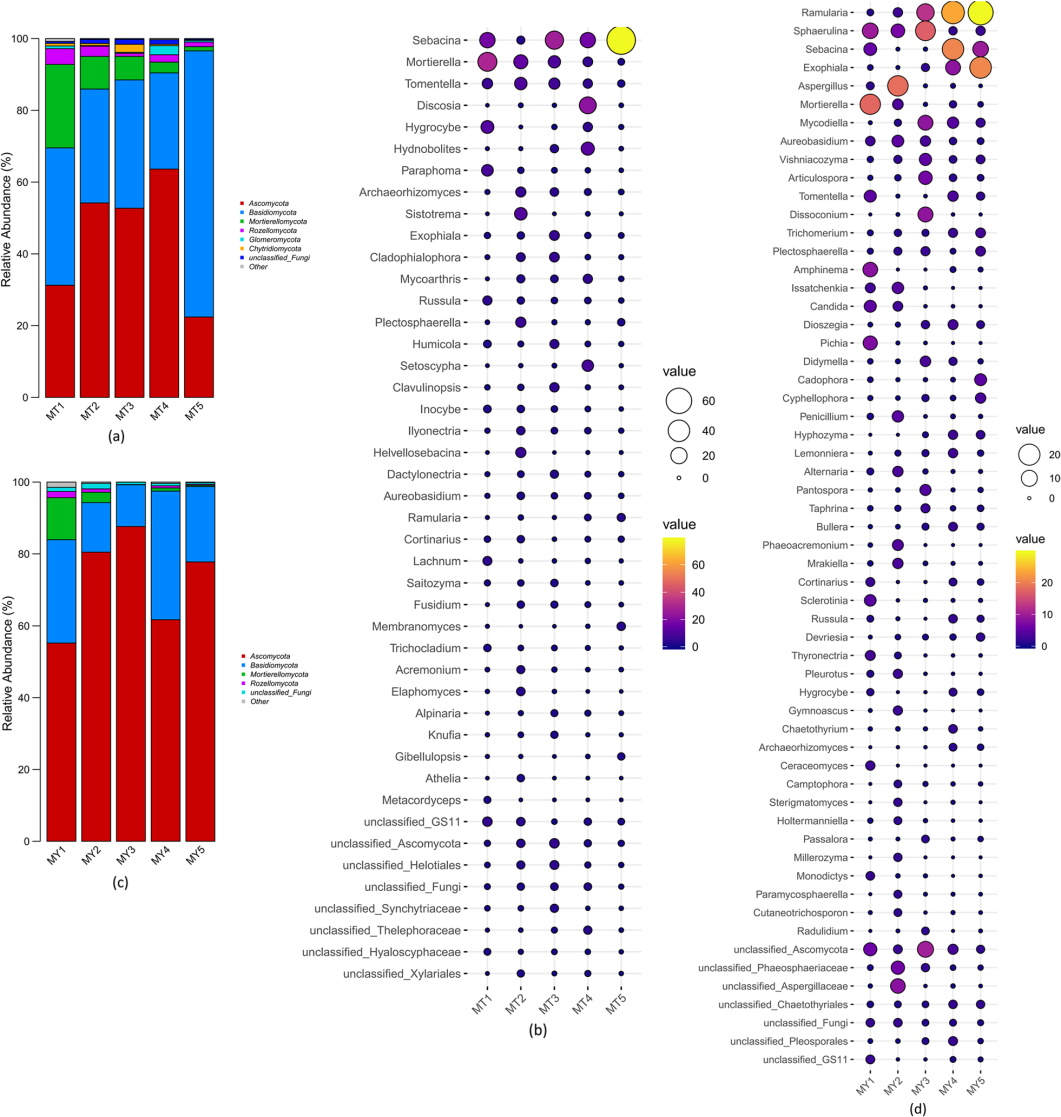
**a** and **b** Relative abundance of fungal species at the phylum (**a**) and genus (**b**) levels in MT groups. **c** and **d** Relative abundance of fungal species at the phylum (**c**) and genus (**d**) levels in MY groups

Figure 3a shows the microbial species composition and relative abundance (mean relative abundance > 1%) at the phylum level in MT groups. Excluding unclassified and others, the five MT groups were mainly composed of fungi belonging to the phyla *Ascomycota, Basidiomycota, Mortierellomycota. and Rozellomycota.,* with the former three phyla representing the core fungal communities. Although *Mortierellomycota* was dominant at all five stages, its relative abundance gradually decreased with the extension of the *Epimedium koreanum *Nakai growth period. In MT1 and MT5, *Basidiomycota* was the most dominant phylum (38.29% and 74.18%, respectively), followed by *Ascomycota* and *Mortierellomycota*. In MT2, MT3 and MT4, *Ascomycota* was the most dominant phylum, followed by *Basidiomycota* and *Mortierellomycota*. In addition, *Chytridiomycota* was the only phylum with relative abundance greater than 1% in MT3, whereas the relative abundance of the other phyla was less than 1%. *Glomeromycota* was the dominant phylum at the MT4 stage.

Figure 3b shows the microbial species composition and relative abundance (mean relative abundance > 1%) at the genus level in MT groups. Excluding unclassified and others, the five MT groups were mainly composed of *Sebacina sp.*, *Mortierella sp.*, *Tomentella sp.*, *Discosia sp.* and *Hygrocybe sp.*, and the core genera were *Sebacina sp.*, *Mortierella sp.* and *Hygrocybe sp*. In MT1 and MT2, *Spore* was the most dominant genus, followed by *Sebacina sp.*, *Hygrocybe sp.*,*Sistotrema* sp. and *Paraphoma sp.*In MT3 and MT5, *Sebacina sp.* was the most dominant genus (accounting for 64.08% in MT5), followed by *Mortierella sp.* and *Tomentella sp. Discosia* sp.(13.93%) was the most dominant genus in MT4, followed by *Sebacina sp.* (10.81%), *Hydnobolites sp.* (7.16%) and *Setoscyph sp.* (4.89%).

Figure 3c shows the microbial species composition and relative abundance (mean relative abundance > 1%) at the phylum level in MY groups. Excluding unclassified and others, the five MY groups were mainly composed of *Ascomycota*, *Basidiomycota*, and *Mortierellomycota*, and *Ascomycota* and *Basidiomycota* were the core phyla. *Ascomycota* was the most dominant phylum, followed by *Basidiomycota. Mortierellomycota* was the only phylum whose relative abundance was higher than 1% in MY1 and MY2. *Rozellomycota* was dominant in MY1.

Figure 3d shows the microbial species composition and relative abundance (mean relative abundance > 1%) at the genus level in MY groups. Excluding unclassified and others, the five MY groups were mainly composed of the genera *Ramularia*, *Sphaerulina sp.*, *Sebacina sp.*, *Exophiala sp.*, *Aspergillus sp.*, *Mortierella sp.* and others, and the core genus was *Glomus sp..* In MY1, *Mortierella* sp.(11.61%) was the most dominant genus, followed by *Glomus* (6.27%) and *Amphinema* (5.22%). In MY2, *Aspergillus sp.* (15.22%) was the most dominant genus, followed by *Glomus* (5.60%) and *Aureobasidium sp.* (3.80%). In MY3, *Glomus* sp. (13.34%) was the most dominant genus, followed by *Columella sp* (9.78%), *Dissoconium sp.* (6.78%) and *Mycodiella sp.* (6.72%). In MY4 and MY5, *Streptospira sp.* was the most dominant genus, followed by *Cerulococci sp.* and *Trichoderma sp.*. *Amphinema sp.*, *Aspergillus sp.*, *Dissoconium sp.* and *Articulospora sp.* were the dominant fungal genera in MY1, MY2 and MY3 groups.

### Analysis of different flora and differentially expressed genes (DEGs)

**Fig. 4:**
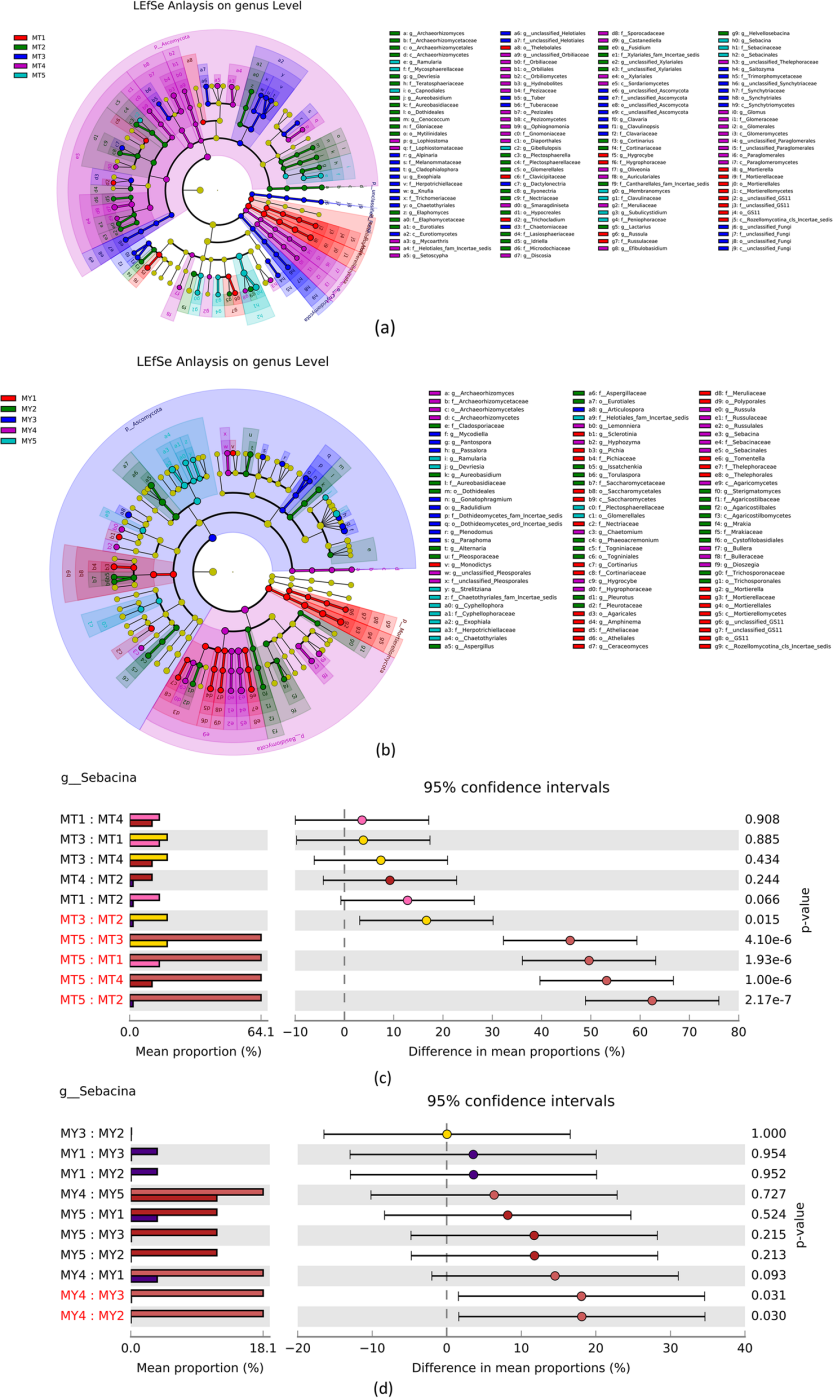

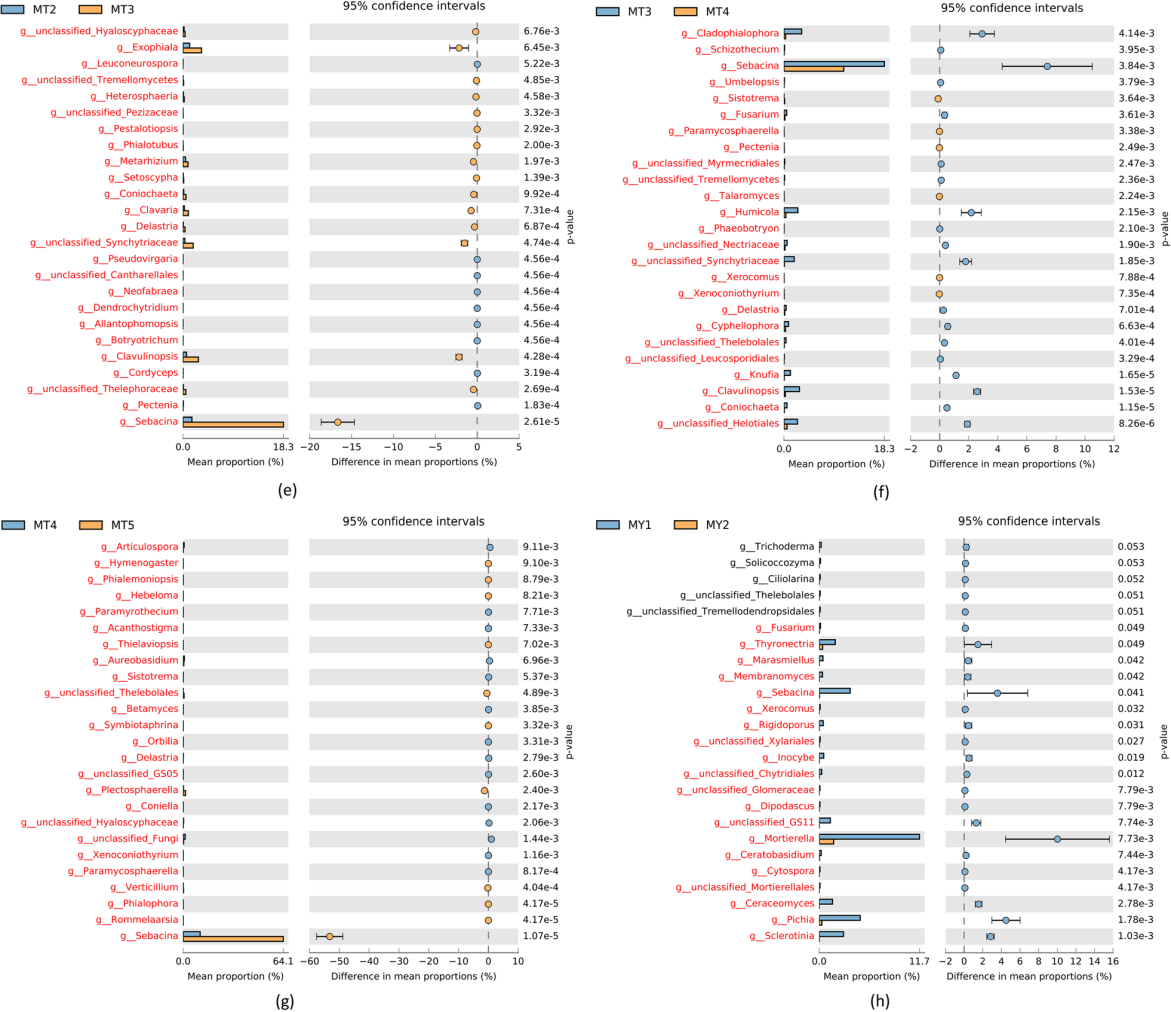
**a** and **b** LEfSe of MT (**a**) and MY (**b**) groups. c and d ANOVA plots of *Sebacina sp.* in MT (**c**) and MY (**d**) groups. **e**–**g** Plots of group differences between MT2–5 respectively. **h** Plot of group differences in *Sebacina s*genus level MY1-2

LEfSe is used to determine the genetic or functional features that best explain the differences between two or more groups of samples under different biological conditions or environments as well as the degree to which these features influence the differences between groups. Our results showed that *Mortierella sp.*, *Hygrocybe sp.* and *Russla sp.* were the main fungal communities at the MT1 stage; *Plectosphaerella sp.*,*Archaeorhizomyces sp.*,and *HelvelloSebacina sp.*at the MT2 stage; *Cladophialophora sp., Knufia* and *Exophiala* at the MT3 stage; *Mycoarthris sp.,Discosia sp.*, *Hydnobolites sp.*, and *Setoscypha sp.* at the MT4 stage; and *Ramularia*, *Sebacina sp.*, and *Gibellulopsis sp.* at the MT5 stage (Fig. 4a).The species richness of *Sebacina sp.* was significantly higher in MT3 than in MT1 and MT2. and decreased slightly from MT4.The MT5 group showed the highest species richness of *Sebacina sp.* (Additional file 1: Table S3).

*Mortierella sp., Amphinema sp. Pichia sp., Sclerotinia sp., Ceraceomyces sp., Monodictys sp.*and *Tomentella sp.* were the main fungal communities at the MY1 stage; *Alearnaria sp.*, *Pheurotus sp. Aureobasidium sp.* and *Aspergillaceae* sp.at the MY2 sage; *Mycodiella sp.*, *Plenodomus sp.,Paraphoma sp.*and *Articulospora sp.* at the MY3 stage;*Russula sp.*,*Sebacian sp.*, *Hyphozym*a sp. and *Agaricomycetes sp.*at the MY4 stage; and *Ramularia sp.*, *Peviresia sp.* and *Cyphellophoraceae sp.* at the MY5 stage (Fig. 4b). The species richness of *Sebacina sp.* was significantly higher in MY4 than in MY1, MY2 and MY3. and decreased slightly from MT5.The MT4group showed the highest species richness of *Sebacina sp.* (Additional file 1: Table S3).

DESeq2 is commonly used to analyze DEGs identified in transcriptome data. As an extension, this method is often used to analyze the taxonomic sequencing data for determining the differential abundance of microbial communities between two datasets (Additional file 1: Table S4). Figure 4c–g shows the differential gene analysis of *Secabina* sp.in MT samples. Comparison between MT1 and MT2 groups showed that the sum of species richness of *Sebacina sp.* was 26,302 in MT1 and 1,943 in MT2. After standardization, the mean abundance of *Sebacina sp.* was 5704.719, and and Sebacina sp. abundance in MT2 was fourfold higher than that in MT1. Similarly, comparison between MT1 and MT3 stages showed that the sum of species abundance of *Sebacina sp.* was 20,385 at the MT3 stage, and the mean abundance after standardization was 8626.862. The difference multiple in MT3 was approximately 0.5-fold higher than that in MT1. In the MT4 vs. MT5 comparison, the sum of species abundance of *Sebacina sp.* was 14,576 in M4 and 178,449 in MT5. After standardization, the mean abundance was 31,564.497, and the difference multiple in MT5 was approximately threefold higher than that in MT4.

The differential gene analysis of *Secabina* sp. in MY samples is shown in Fig. 4h. Comparison between MY1 and MY2 groups showed that the sum of the relative richness of MY1 and MY2 species was 124 and 12, respectively. After standardization, the mean abundance of MT1 was 36.482, and MT1 was fourfold higher than that of MY2. The MY1 vs. MY3 comparison showed that the sum of the relative richness of MY3 species was 27, and the mean abundance after standardization was 55.080, which was fivefold higher than that of MY3. The MY4 vs. MY5 comparison revealed that the sum of relative richness of MY4 and MY5 species was 4,292 and 4,766, respectively. After standardization, the mean abundance was 1534.501, which was 0.25-fold higher than that of MY4.

### Relationship between fungal communities and environmental factors

VIF analysis is a commonly used to screen environmental and clinical factors. The larger the VIF value, the more serious the multicollinearity relationship between the independent variables. Environmental factors with VIF values greater than 10 are generally considered useless. Therefore, in this study, the environmental factors with VIF > 10 were discarded. After multiple screening, six environmental factors, including soluble salt, total nitrogen, alkali-hydrolyzed nitrogen, total phosphorus, total potassium and available potassium, were found to be useful factors. The R 3.6.0 software was used to analyze the Spearman correlation between the rhizosphere soil fungi (genus level) and soil physical and chemical properties in MT samples (Additional file 1: Table S5). *Sebacina sp.* showed significant positive correlation with total phosphorus, total nitrogen, alkali-hydrolyzed nitrogen and total potassium, and significant negative correlation with soluble salt and available potassium. *Mortierella* sp.and *Russula* sp.were positively correlated with soluble salt but negatively correlated with total potassium, total nitrogen, alkali-hydrolyzed nitrogen, total phosphorus and available potassium. *Cortinarius* sp.showed a significant positive correlation with soluble salt, total potassium and total phosphorus, and significant negative correlation with total nitrogen, available potassium and alkali-hydrolyzed nitrogen.

Spearman correlation analysis between the endophytic fungi of *Epimedium koreanum *Nakai (genus level) and soil physical and chemical properties of MY samples is shown in Additional file 1: Table S6. *Sebacina sp.* and *Russula* sp.were positively correlated with total potassium, total nitrogen, total phosphorus and available potassium but negatively correlated with soluble salt and alkali-hydrolyzed nitrogen. *Mortierella sp.* was positively correlated with total potassium, soluble salt and total phosphorus but negatively correlated with total nitrogen, alkali-hydrolyzed nitrogen and available potassium. *Cortinarius sp.* was positively correlated with total nitrogen, total phosphorus and total potassium but negatively correlated with soluble salt, alkali-hydrolyzed nitrogen and available potassium.

## Discussion

### Fungal genera beneficial to *Epimedium koreanum *Nakai

Thirty fungal genera with significant differences were identified by LEfSe analysis (Fig. 4). Of these, 17 were beneficial fungi to *Epimedium koreanum *Nakai, 6 were harmful fungi, and 7 had not been previously reported. One genus of harmful fungi was found by GraPhlAn analysis (Fig. 3). The genus *Sebacina* is the core dominant genus in the MT5 and MY4 stages, and the highest content of *Sebacina sp*. was reached in the MT4 and MY5 stages. Previous studies have shown that the genus *Sebacina* has a life-promoting effect on medicinal *Dendrobium officinale *[28]. This means that the key genus *Sebacina* promotes the growth of *Epimedium koreanum *Nakai in late growth stages. *Russula sp.* and *Tomentella sp.* may possess decomposition abilities for complex soil organic matter [29, 30]. *Amphinema sp.* did not negatively affect host plant N uptake but rather elevated the levels of other nutrients and perhaps promoted growth, hence diluting the N pool [31]. *Russula* was the dominant genus during MT1.*Amphinema* was the dominant genus during MY1. The two genera decompose soil organic matter in the early stages of *Epimedium koreanum *Nakai growth to deliver the nutrients needed for its growth. In a report of the rhizosphere of tea, *Discosia sp.* was shown to have the potential to promote plant growth in a variety of plants. For example, the tea rhizosphere was tested on chickpea, maize and pea. [32]

*Aspergillaceae sp*. are known for their phenotypic diversity, including their extremotolerance [33–36] and ability to grow on various carbon sources [35, 37]. Numerous studies have documented the beneficial properties of avirulent *Trichoderma sp.* Strains, which are used for plant protection, biostimulation, and biofertilization. Fungal species belonging to *Mortierellaceae* are important saprobic organisms that live on a wide range of organic substrates, such as soil, plant debris, and animal dung [38–40]. Recent studies on the soil microbiota on a global scale reported *Mortierella sp.* as a key player in the soil core microbiome [41, 42]. Few studies have reported the biotechnological application of *Mortierella sp.* in the food industry for fatty acid production [43–45]. Currently, our research group is investigating the birth-promoting action mechanism and food additives of species of the dark greenwood genus that have been isolated and purified.

There are other beneficial divergent genera, such as those with properties of growth regulation: *Articulospora sp., Sistotrema sp., Rhododendron sp., Exophiala sp., Knufia sp., Pichia sp., and Cortinarius sp*. The latest results in 2022 show that *Articulospora sp.* could provide various benefits in regards to the mobilization of organic or inorganic compounds [46]. *Sistotremab sp*. are involved in the genetic regulation of critical stages of plant development [47]. *Exophiala sp.* [48] and Knufia sp. [47, 49–53] are significant degraders of organic organisms. The genus Rhododendron exhibited antibacterial effects against gram-positive bacteria [54]. *Pichia sp.* was demonstrated to be a promising probiotic for poultry. The results of the latest study showed that [55] *Cortinarius* is an important fungal genera with immense species richness. These beneficial genera provide the basis for the subsequent isolation and study of beneficial endophytes in *Epimedium koreanum *Nakai.

### Fungal genera that are harmful to plants

LEfSe analysis showed that the genera present in MT2 was *Plectosphaerella* and that in MY3 was *Paraphom*a (Fig. 4). *Plectosphaerella sp*. are well known as pathogens of several plant species, causing fruit, root and collarrot collapse [56]. *Paraphoma sp.* are fungal pathogens recently reported to cause alfalfa root rot in Inner Mongolia, China [57]. *Plectosphaerella.* and *Paraphoma* may be the major pathogenic genera of the early growth stage of *Epimedium koreanum *Nakai*.* The GraPhlAn analysis (Fig. 3) indicated the presence of *Sphaerulina sp.* during all periods of MY. Many Populus species and hybrids are susceptible to leaf and stem diseases caused by *Sphaerulina* species [58–60]. Thus, *Sphaerulina sp.* may be the main cause of illness in *Epimedium koreanum *Nakai*.*

LEfSe analysis showed that species of the *Gibellulopsis* genera were present in MT5 and species of the genera *Cyphellophora* were present in MY5 (Fig. 4). The genus *Gibellulopsis* contains only one valid species, *Gibellulopsis nigrescens* [61]. This species was also isolated from a soil sample and was shown to be the cause of wilt of sugar beets in China [62, 63]. The genus *Cyphellophora* has three new species, one of which, *Cyphellophora artocarpi*, may be a sooty blotch and flyspeck pathogen of apple [64]. The common pathogenic fungal genus during the MT5 and MY5 periods was *Ramularia. Ramularia* collo-cygni can remain in an asymptomatic state for several weeks, but after a long period of latent development, it can then undergo a developmental switch to become an aggressive necrotrophic pathogen [65]. As the agent of a late season disease, the shift of *Ramularia collo-cygni* from endophyte to pathogen has been associated with changes in host development from the vegetative to reproductive stages and with a decline in the host antioxidant system during monocarpic senescence [66]. This indicates that in the later stage of growth, *Ramularia* may cause *Epimedium koreanum *Nakai plant disease through variation.

No studies have reported the fungal genera *Archaeorhizomyces, Setoscypha, Sygrocybe, Hygrocybe., Peviresia, Cerulococci, Mycodiella or Streptospira.*

## Conclusion

Li et al. [67] suggested that subtropical tree phyllosphere microbial communities vary with host species identity, plant traits and seasonality. Endophytic isolates identified by Bziuk et al. [68] were affiliated with members of the core seed microbiome, and many of them showed beneficial plant properties. Olimi et al. [69] mentioned that a deep understanding of plant microbiome assembly could lead to the development of potential postharvest biocontrol agents. Research with insights into the *Epimedium koreanum *Nakai rhizosphere soil and leaf endophytic fungal communities could provide the basis for the later isolation of the beneficial microorganisms of cultivable *Epimedium koreanum *Nakai. Alpha diversity analysis of species in *Epimedium koreanum *Nakai showed that the relative abundance of soil fungi in the rhizosphere was higher than that of leaf endophytes during early growth, but the overall sum was basically equal. LEfSe analysis, ANOVA and SMART analysis showed that different *Sebacina sp.* can move between each other in the rhizosphere of soil fungi and leaf endophytes. Nitrogen-phosphorus-potassium elements in the environment have a clear positive effect on the relative abundance of the genus *Sebacina*. In the future, fungi of *Sebacina sp.* can be further cultivated to study the quality of *Sebacina sp*. in promoting the growth of *Epimedium koreanum *Nakai. Also, pathogenic bacteria can be studies in regards to early pest control and better cultivation and harvesting of *Epimedium koreanum *Nakai.

## Supplementary Information

**Additional file 1:** Results Analysis table of Epimedium Koreanum Naikai.

## Data Availability

The data that supports the fingdings of this study as available in the supplementary material of this article.

## Abbreviations

MT1: Rhizosphere soil samples during the seedling stage
MT2: Rhizosphere soil samples during the delayed seedling stage
MT3: Rhizosphere soil samples during vigorous growth boom
MT4: The rhizosphere soil samples during the flowering stage
MT5: The rhizosphere soil samples during the dormant period
MY1: Leaf samples during the seedling stage
MY2: Leaf samples during the delayed seedling stage
MY3: Leaf samples during vigorous growth boom
MY4: Leaf samples during the flowering stage
MY5: Leaf samples during the dormant period
