# Supplementary material for: Diversity analysis of leaf endophytic fungi and rhizosphere soil fungi of Korean Epimedium at different growth stages
Source: Environ Microbiome. 2022 Oct 21;17:52. doi: 10.1186/s40793-022-00446-w (PMC9585767; doi:10.1186/s40793-022-00446-w)
Supplement: Supplementary file 1 — Additional file 1: Results Analysis table of Epimedium Koreanum Naikai. [file 40793_2022_446_MOESM1_ESM.docx]

**Additional information**

**Table S1.** The alpha diversity index of the rhizosphere soil fungi

| Sample | Shannon | Chao | Ace | Simpson | Shannoneven | Coverage |
| --- | --- | --- | --- | --- | --- | --- |
| MT1-1 | 3.259999 | 1338.068027 | 1306.437336 | 0.126055 | 0.468431 | 0.994878 |
| MT1-2 | 4.572659 | 1730.286957 | 1754.286205 | 0.03726 | 0.627934 | 0.995035 |
| MT1-3 | 4.158218 | 1701.366337 | 1693.355003 | 0.058046 | 0.577317 | 0.994005 |
| MT2-1 | 5.143191 | 1982.014151 | 1986.8017 | 0.017614 | 0.706481 | 0.988133 |
| MT2-2 | 5.355329 | 2241.562212 | 2155.753073 | 0.016884 | 0.723812 | 0.987365 |
| MT2-3 | 4.903273 | 2019.566964 | 2004.152137 | 0.049744 | 0.669435 | 0.987476 |
| MT3-1 | 5.032947 | 1629.47644 | 1648.392963 | 0.02308 | 0.703917 | 0.989396 |
| MT3-2 | 4.977719 | 1583.62069 | 1627.375225 | 0.024646 | 0.694754 | 0.991537 |
| MT3-3 | 4.957122 | 1645 | 1644.87496 | 0.027508 | 0.693008 | 0.989817 |
| MT4-1 | 5.364785 | 2101 | 2040.924479 | 0.023824 | 0.723429 | 0.987407 |
| MT4-2 | 5.382906 | 2207.897436 | 2153.435475 | 0.021262 | 0.71922 | 0.990341 |
| MT4-3 | 4.511817 | 2443.011583 | 2412.024414 | 0.113621 | 0.600043 | 0.989565 |
| MT5-1 | 3.368136 | 2593.003247 | 2606.550723 | 0.181827 | 0.44371 | 0.993312 |
| MT5-2 | 3.444333 | 2558.086957 | 2486.259329 | 0.17932 | 0.454478 | 0.99386 |
| MT5-3 | 3.588498 | 2691.378571 | 2638.477285 | 0.165053 | 0.472551 | 0.993202 |

**Table S2.** The alpha diversity index of the endophytic fungi of *Epimedium koreanum Nakai*

| Sample | Shannon | Chao | Ace | Simpson | Shannoneven | Coverage |
| --- | --- | --- | --- | --- | --- | --- |
| MY1-1 | 4.774513 | 547.584416 | 797.409491 | 0.028109 | 0.817872 | 0.861908 |
| MY1-2 | 5.043697 | 536.458333 | 581.145954 | 0.014048 | 0.872035 | 0.817898 |
| MY1-3 | 4.895958 | 598.264706 | 651.564816 | 0.017049 | 0.841218 | 0.839422 |
| MY2-1 | 3.38749 | 289.428571 | 351.333333 | 0.046206 | 0.735584 | 0.997281 |
| MY2-2 | 3.418738 | 289.363636 | 454.691505 | 0.166118 | 0.667206 | 0.888476 |
| MY2-3 | 4.300412 | 642.535714 | 649.012479 | 0.04399 | 0.685557 | 0.988396 |
| MY3-1 | 4.16339 | 876 | 871.230863 | 0.038019 | 0.64403 | 0.991096 |
| MY3-2 | 4.443577 | 439.576923 | 474.367593 | 0.03011 | 0.756001 | 0.97127 |
| MY3-3 | 3.930494 | 749.725 | 755.350018 | 0.067206 | 0.626586 | 0.98612 |
| MY4-1 | 4.408979 | 1568.057143 | 1627.389006 | 0.06676 | 0.638173 | 0.968713 |
| MY4-2 | 4.971724 | 1605.732824 | 2169.157682 | 0.025505 | 0.736432 | 0.932991 |
| MY4-3 | 4.858101 | 1338.853659 | 1896.110815 | 0.024074 | 0.735185 | 0.936308 |
| MY5-1 | 3.696809 | 1946.905 | 2562.211658 | 0.127353 | 0.522582 | 0.978672 |
| MY5-2 | 3.899714 | 2056.111111 | 2701.250685 | 0.088637 | 0.551995 | 0.979673 |
| MY5-3 | 4.895509 | 1646.877863 | 2587.567391 | 0.028237 | 0.72692 | 0.92808 |

**Table S3.** Sebacina sp. result values at a LDA> 4

| LEfSe(LDA>4)  MT | MT1 | MT2 | MT3 | MT4 | MT5 |
| --- | --- | --- | --- | --- | --- |
|  | 5.3576 | 1.2445 | 17.2772 | 9.44722 | 61.6209 |
| LEfSe(LDA>4)  MY | MY1 | MY2 | MY3 | MY4 | MY5 |
|  | 5.0977 | 0.0841 | 0.0073 | 19.2569 | 22.9831 |

**Table S4.** The Sebacina genus level taxonomic sequencing data between two datasets

|  | baseMean | log2Fold Change | pvalue | padj |
| --- | --- | --- | --- | --- |
| MT1 VS MT2 | 5704.719261 | -4.093867733 | 2.670093526 | 0.00004175 |
| MT1 VS MT3 | 8626.861726 | -0.52848006 | 0.492867663 | 0.64643177 |
| MT4 VS MT5 | 31564.49718 | 3.574734268 | 6.376274801 | 1.721594196 |
| MY1 VS MY2 | 36.48278617 | -4.93880021 | 0.020230523 | 0.88143145 |
| MY1 VS MY3 | 55.08079541 | -5.125415693 | 1.486888479 | 3.45415631 |
| MY4 VS MY5 | 1534.501113 | -0.254834999 | 0.557687643 | 0.97272707 |

**Table S5.** The Spearman correlation EnvCorr between rhizosphere soil fungi (genus level)

| the rhizosphere soil fungi | soluble salt | total nitrogen | alkali-hydrolyzed nitrogen | total phosphorus | total potassium | available potassium |
| --- | --- | --- | --- | --- | --- | --- |
| *Sebacina* | -0.421428571 | 0.387846446 | 0.500000000 | 0.112600581 | 0.55992934 | -0.25000000 |
| *Mortierella* | 0.678571429 | -0.965147838 | -0.464285714 | -0.711349703 | -0.368515795 | -0.332142857 |
| *Russula* | 0.585714286 | -0.759607095 | -0.414285714 | -0.453976946 | -0.330948651 | -0.071428571 |
| *Cortinarius* | 0.400000000 | -0.171581838 | -0.010714286 | 0.246648892 | 0.368515795 | -0.535714286 |

**Table S6.** The Spearman correlation EnvCorr between endophytic fungi of (genus level) in endophytic fungi samples

| the endophytic fungi of Epimedium | soluble salt | total nitrogen | alkali-hydrolyzed nitrogen | total phosphorus | total potassium | available potassium |
| --- | --- | --- | --- | --- | --- | --- |
| *Sebacina* | -0.775 | 0.566577527 | -0.221428571 | 0.652368446 | 0.704831181 | 0.1 |
| *Mortierella* | 0.060714286 | -0.348525608 | -0.585714286 | 0.112600581 | 0.547406958 | -0.435714286 |
| *Cortinarius* | -0.461279981 | 0.104602182 | -0.549571852 | 0.344465807 | 0.692256312 | -0.158564993 |
| *Russula* | -0.578401851 | 0.412096528 | -0.401818108 | 0.631220065 | 0.673302749 | 0.00360375 |

**Additional files list**

**Table S1.** The alpha diversity index of the rhizosphere soil fungi

**Table S2.** The alpha diversity index of the endophytic fungi of *Epimedium koreanum Nakai*

**Table S3.** Sebacina sp. result values at a LDA> 4

**Table S4.** The Sebacina genus level taxonomic sequencing data for the differential abundance of microbial communities between two datasets

**Table S4.** The Spearman correlation EnvCorr between rhizosphere soil fungi (genus level) and soil physical and chemical properties in rhizosphere soil fungi samples

**Table S5.** The Spearman correlation EnvCorr between endophytic fungi of(genus level) and soil physical and chemical properties in endophytic fungi of samples
